# Role of Deep Learning in Computed Tomography

**DOI:** 10.7759/cureus.39160

**Published:** 2023-05-17

**Authors:** Yash Garg, Karthik Seetharam, Manjari Sharma, Dipesh K Rohita, Waseem Nabi

**Affiliations:** 1 Internal Medicine, Wyckoff Heights Medical Center, New York, USA

**Keywords:** ai and machine learning, coronary computed tomoangiography, intervention cardiology, artificial intelligence in radiology, computed tomography (ct )

## Abstract

Computed tomography has played an instrumental role in the understanding of the pathophysiology of atherosclerosis in coronary artery disease. It enables visualization of the plaque obstruction and vessel stenosis in a comprehensive manner. As technology for computed tomography is constantly evolving, coronary applications and possibilities are constantly expanding. This influx of information can overwhelm a physician's ability to interpret information in this era of big data. Machine learning is a revolutionary approach that can help provide limitless pathways in patient management. Within these machine algorithms, deep learning has tremendous potential and can revolutionize computed tomography and cardiovascular imaging. In this review article, we highlight the role of deep learning in various aspects of computed tomography.

## Introduction and background

As cardiovascular imaging data is becoming more multi-dimensional in nature and increasingly complex, this will pose significant difficulties in the healthcare sector in years to come [1]. Similarly, diagnostic modalities are progressively evolving with the addition of various new parameters [2]. These technological advancements have had significant impacts in the field of computed tomography (CT) [3,4]. In parallel with the growth of cardiovascular imaging, there is a massive influx of data arising from a multitude of wearable devices and smart apps, which will inevitably integrate with clinical management [4]. Similarly, the technology behind electronic medical record systems is also growing and is capable of collecting and storing more patient information [5]. With a plethora of information arising from multiple user interfaces, this in turn may become more of a curse rather than a blessing for any healthcare personnel [6]. Furthermore, data being exceedingly complex will supersede the capabilities of current statistical software [7].

To navigate through this complex labyrinth of information, artificial intelligence (AI) is becoming a vital necessity for aggregating information in a clinically meaningful manner [4,5]. AI can interpret information and lead to data-driven discoveries typically not seen with traditional statistics [8,9]. Within the various algorithms of AI, deep learning has the most revolutionary potential and is at the forefront of AI in cardiovascular imaging [6,10]. With rapid advances in graphical processing units (GPU) and emergence of cloud technology, this has greatly propelled the growth of deep learning [3]. Deep learning enables image classification and segmentation in imaging [11]. Among non-invasive modalities, computed tomography angiography (CTA) is an emerging modality with efficacy and accuracy paralleling other contemporary modalities. Furthermore, CTA can clearly exclude obstructive coronary artery disease (CAD) with high negative predictive value [12]. Deep learning can automate redundant tasks and augment diagnostic or prognostic capabilities of CTA [4,11]. In this review article, we highlight the role of deep learning in various aspects for computed tomography. 

## Review

Types of machine learning

Machine learning (ML) is an umbrella term which encompasses a wide variety of algorithms [1,4]. These can be broadly categorized into supervised learning, unsupervised learning, semi-supervised learning, and reinforcement learning [1,4]. Supervised learning uses annotations and labels within a dataset [5]. Unsupervised learning is independent and does not requires any labels or classes within a dataset [7]. Semi-supervised learning uses properties present within supervised and unsupervised learning [4]. It can comprehend data with or without labels. Lastly, reinforcement learning is similar to human psychology [3]. It utilizes certain reward criteria for the algorithm to function independently.

One of the weaknesses of current statistics is its prediction is not a strong suit of these computational programs [8,13]. Furthermore, current statistical software cannot handle large or complex information [1,7]. For this particular aspect, ML algorithms are strikingly different [3,7]. This is particularly applicable to deep learning. As data becomes larger, it helps ML algorithms to function better, and accuracy improves [11]. Machine learning can unravel hidden new relationships, not possible with conventional statistics [5].

Overview of deep learning

Among the variety of algorithms present, deep learning (DL) algorithms have the most revolutionary potential [14]. “Deep” technically refers to a multilayered separation [11]. DL is being widely utilized in various parts of human industry from information technology to the commercial industry [2]. For example, DL framework is being utilized in self-driving cars to voice recognition software from Google and Amazon [2,3,13]. The architecture of DL is similar to human neuronal structure [4]. DL algorithms can process and extract information in a series of hierarchical layers [15]. It has proven to be a valuable asset in image classification, speech recognition, genomics, and image segmentation. 

DL vastly differs from contemporary ML algorithms [2]. Supervised and unsupervised learning algorithms generally require longer training and experience for achieving acceptable results and accuracy [15]. Intense preparation may be required for the algorithm to function properly. In contrast, the accuracy of deep learning algorithms is easier to improve by augmenting the training dataset or elevating the network capacity [15]. They require substantially lesser domain knowledge to perform a function.

Architecture of deep learning

DL encompasses a wide number of algorithms designed to perform a variety of algorithms [11]. In Fully Connected Networks (FNNs), every unit in any layer is linked to every unit in the succeeding and preceding layers [16]. For Deep Belief Networks (DBNs), each layer contains statistical dependencies corresponding to units in the prior layers [17]. Convolutional Neural Networks (CNNs) consist of a segment that allows hierarchical feature interpretation and another segment performing classification or regression [11]. Autoencoders (AE) contain encoding and decoding parts which connect the input to the output which provides valuable properties within the data [18]. Recurrent Neural Networks (RNNs) utilize internal states through feedback loops to comprehend the input [11]. Within RNN, long short term memory and gated recurrent unit are commonly used algorithms.

Among the DL algorithms, CNNs are frequently used in cardiovascular imaging. CNNs prioritize feature optimization while examining the data through the convolutional layers. The classification layers are located in the last layers of the CNN. In addition, feature maps can be produced by convolutional layers from various parameters. Visual Geometry Group (VGC) is a simple CNN framework with lesser depth [19]. GoogleNet [20] and ResNET [21] have CNN-like structures with multiple layers of complexity.

The stacked Denoised AE is a frequently used AE algorithm [18]. This algorithm can create clean input from corrupted input. The U-net is another AE-like framework that can associate layers of the encoder with the decoder [22]. The U-net plays an important role in the segmentation of images in cardiology.

Emerging relationship between computed tomography and machine learning

CAD is the leading cause of mortality in the developed world [23]. For the evaluation of CAD, cardiac CT is non-invasive and is a well-established modality. Cardiac CT depicts the extent of the atherosclerotic process in the coronary artery tree [24]. With the advent of CTA, it has rapidly shifted diagnostic paradigms in the assessment of CAD [25,26]. Cardiac CTA has a negative predictive value approaching 100%, and the sensitivity and specificity reach 98% and 89% respectively. Without question, cardiac CT can reliably exclude CAD in suspected cases. As a result, cardiac CT is integrated into many diagnostic algorithms in patients with obstructive CAD in clinical or emergency settings [26]. 

Cardiac CT provides a comprehensive portrait of CAD from the level of atherosclerotic plaque to functional evaluation of various coronary lesions [24]. Many prestigious societies are firmly advocating cardiac CT as a first-line option for suspected CAD [27,28]. As the technology continues to thrive and evolve, there will be an influx of new parameters [9]. These new parameters may provide additional information but not in a meaningful manner. There is a growing interest within the cardiovascular community in the integration of ML algorithms with cardiac CT and other imaging modalities [4,10]. ML algorithms can streamline the clinical workflow and help provide more time for clinical decision-making [29,30]. They can execute mundane tasks and help analyze medical information in a clinically meaningful manner [31]. The integration of ML algorithms with cardiac CT will pave the pathway for precision medicine in cardiovascular imaging [13].

Role of deep learning in coronary artery calcium assessment

Non-contrast coronary artery calcium (CAC) evaluation is an important facet of coronary CT [32]. CAC scoring helps assess the extent of atherosclerotic disease [33] and is a paramount parameter for cardiovascular risk stratification. There is an inherent relationship between elevating CAC and the presence of obstructive CAD [32]. A number of studies have explored the role of deep learning for CAC in cardiac CT.

Lessman et al. utilized a CNN framework to evaluate CAC [34]. The CNN algorithms created a bounding box around the heart. This bounding box corresponds to Hounsfield units. If patients were above certain thresholds for Hounsfield units, they were considered to be candidates for CAC by cardiac CT. Based on this score, patients were segregated into five risk categories. Similarly, Lessman et al. utilized a CNN algorithm for the identification of calcification in low-dose CT [35]. Wolternick et al. assessed the role of a multi-layered CNN architecture for measuring CAC which did not need coronary extraction [36]. The CNN algorithm created a bounding box around the heart in multiple planes and another CNN simultaneously detected CAC. The algorithm obtained a high correlation coefficient of 0.95.

Cano-Espinosa et al. created a CNN algorithm that automatically generated Agaston score for CAC in 5973 CT images [37]. Surprisingly, Cano-Espinosa et al. achieved a Pearson correlation coefficient of 0.932. Santini et al. used a multi-layered CNN algorithm for classification of coronary lesions with various CT volumes [38]. Their CNN algorithm was able to reveal a Pearson correlation of 0.983.

Role of deep learning in image segmentation for computed tomography

One of the primary uses of cardiac CT in clinical practice is segmentation of various cardiac structures. Cardiac CT and CTA require manual segmentation of structures which can be time-consuming [30] and may diminish time for clinical management. Automated deep-learning algorithms can be particularly useful for producing rapid and reliable results [9]. It could set the platform for automated segmentation in the near future.

Liu et al. utilized a multi-layered FCN for automatic segmentation of the left atrium for 3D CT volumes [39]. The authors further improved the algorithm to reach a Dice index of 93%. Hong et al. evaluated a DBN algorithm for segmentation and classification of abdominal aortic aneurysm from CT [38,39]. López-Linares et al. explored the potential of CNN framework for evaluation of aortic thrombus [40]. Automatic segmentation was performed for preoperative and postoperative imaging. Jin et al. applied a CNN algorithm to automatically segment the left atrial appendage for diagnosing atrial fibrillation from CTA [41]. In relation to manual annotation, there was a mean Dice overlap of 94.76% and a mean volume overlap of 91.10%. The computation time was less than 40 seconds per volume. Dormer et al. used a CNN algorithm for complete segmentation of the heart, obtaining an overall accuracy of 87.2% ± 3.3% and an overall chamber accuracy of 85.6 ± 6.1% [42].

Baskaran et al. explored the potential of deep learning in automatic segmentation of cardiac structures on CT [30]. They used a U-Net deep learning algorithm to automatically segment ten structures which included various structures in the left and right sides of the heart along with the great vessels. The overall Dice score was 0.932 and results were consistent across various subsets. Strikingly, automatic segmentation took an average of 440 seconds per case which greatly contrasts with manual segmentation of five hours.

Role of deep learning in detection of cardiac structures for computed tomography

Cardiac CT is extensively used for identification and detection of various structures. With appropriate training, deep learning algorithms can greatly aid physicians in this process. De Vos et al. utilized a CNN algorithm to identify cardiac and aortic regions in 2D images derived from CT slices and find the corresponding regions on 3D [43]. The algorithm would detect these regions in 3D CT and place a 3D bounding box around the findings. Moradi et al. assessed the role of CNN algorithm to aid automatic segmentation and anatomic recognition. They arranged the various body areas visualized in CT into nine categories, each representing a relevant area relating to a disease or key cardiovascular feature. The CNN algorithm was able to devise a schematic map to a corresponding CT slice and relevant level. Moradi et al. were able to report a margin zero and margin 1 accuracy of 91.7% and 98.8% was effective in matching a CT image to a relative narrow anatomic window [44]. Zreik et al. applied a CNN algorithm for automatic segmentation of the left ventricle from CTA scans in 60 patients [45]. Left ventricular voxel classification was performed by a bounding box around the left ventricle. The algorithm produced a Dice index of 0.85 and a mean absolute surface distance of 1.1 cm. 

Recently, Baskaran et al. explored the role of deep learning algorithms in identification and quantification of cardiovascular structures from CTA [29]. They utilized a U-Net architecture in 166 patients undergoing CTA for assessing left ventricular volume, left atrial volume, right ventricular volume, and right atrial volume, and left ventricular myocardial mass. The combined Dice score was 0.9246. The deep learning architecture correlated with manual annotation for left ventricular volume (r=0.98), right ventricular volume (r=0.97), left atrial volume (r=0.78), right atrial volume (r=0.97), and left ventricular myocardial mass (r=0.94) with statistical significance (p<.05).

Role of deep learning in various aspects of computed tomography

Zreik et al. employed a CNN algorithm to automatically detect coronary artery stenosis in CTA for 166 patients [46]. In relation to invasive fractional flow reserve, the network produced a c- statistic of 0.74 ± 0.02 with accompanying specificities of 77%, 71%, and 59% at sensitives of 60%, 70%, and 80% respectively. Motwani applied a DL algorithm in CTA for 10,300 patients with suspicion of CAD to predict five-year all-cause mortality [47]. Interestingly, the ML framework displayed a higher area under curve compared with Framingham risk scores (0.79 vs 0.61) or CTA severity scores (0.79 vs SSS 0.64, SIS 0.64, DI 0.62) for predicting all-cause mortality (p<.0001). Commanduer et al. utilized a CNN algorithm to evaluate epicardial adipose tissue in CT [48]. Gulsun et al. assessed the role of a CNN algorithm in extracting blood vessel centerlines in CT [49]. 

Pitfalls of deep learning

Although the early results of deep learning appear promising, there are still many significant issues [15]. The “black box” nature of deep learning is not easily understood and can be difficult for clinical interpretation [4]. A number of approaches have been suggested to improve interpretability, but it may require higher costs or larger data. Another common complaint is overfitting [15]. This can occur with smaller data samples or overly intricate algorithms. The choice of algorithm is dictated by the purpose of analysis and size of data. Deep learning may also be associated with some ethical concerns regarding bias or unintentional manipulation of findings [50]. Consistent results need to be maintained while using the same deep-learning algorithm at different academic sites [51]. Results may vary from one center to another with the same algorithm. There is no standardization of deep learning algorithms. The majority of previous studies compare the results of deep learning with the C-statistic and area under the receiver operating curve. There is no clear cut-off mark for c-statistic [52]. Although this is effective, other statistical metrics need to be used to truly evaluate the results of deep learning. 

Potential of deep learning

CT and CTA play an indispensable role in the modern management of obstructive CAD [26] and other cardiovascular entities. Though numerous technological advances have made significant strides in the field of CT, there is a growing concern regarding the integration of multiple parameters and settings in clinical practice [9]. In this current era of clinical care, physicians are facing unprecedented work demands with rigorous time constraints on a daily basis [31]. For each and every patient, cardiologists have to acquire multiple images, processing, perform simple to complex measurements, interpret images, and finally write reports [31]. This can lead to excessive fatigue, diminished attention span, and decreased memory leading to multiple inconsistencies in findings [53]. Manual processes are known to cause substantial inter-observer variability and decreased reproducibility. These trends are likely to worsen as we move forward as technology is consistently evolving and increasing complexity of imaging data [1].

Deep learning offers limitless opportunities and opens new frontiers in the field of CT [29]. In contrast to its ML sibling algorithms, deep learning processes information through a hierarchy of layers [5,7]. The performance of deep learning increases exponentially with larger data sets [15]. Furthermore, deep learning can process a number of raw images without having any prior information in various aspects [11]. The algorithm can extract information from complex data and predict with great accuracy. Deep learning can automate a number of basic tasks and expedite a number of clinical processes [29]. With adequate training, deep learning can demonstrate tremendous accuracy and highly correlate with manual calculations.

It must be emphasized that deep learning will not replace any healthcare personnel but serve as an invaluable extension to any physician [31]. Deep learning algorithms can greatly augment clinical workflows because they can serve as an additional reader [30]. In addition, these algorithms can segment structures and identify abnormal changes in morphology. This can greatly improve clinical interpretation and improve the reproducibility of findings. This can reduce the workload and time required to process images. More time can be diverted to clinical care [31]. Simultaneously, it does not mean a physician has diminished responsibilities but must be cognizant and vigilant of the findings. Physicians need to constantly monitor the output from these algorithms and determine the clinical relevance.

Within deep learning, there are a number of other algorithms which have tremendous potential. This includes the general adversial network (GAN). GAN has the potential of distinguishing between fake images and real images [54]. It has two generators that create real and fake images. It has been used in intra-venous ultrasound and is underutilized in computed tomography. Another deep learning algorithm is the capsule network, which has fewer training requirements than traditional CNN algorithms [11]. Their interpretive prowess of capsule network is more akin to human perception. It is more frequently used in brain tumor and breast cancer classification. 

For deep learning to thrive and prosper in CT or any other field of cardiovascular imaging, a form of universal standard for data standardization may be necessary [5]. Each medical center has its own distinct classifications, protocols, and acquisition methods [5]. If a common method or approach can be developed, this can facilitate the growth of deep learning and other ML approaches. Since deep learning has a requirement for large data, some form of data sharing is required among institutions. This can excessively time-consuming and tedious process due to regulations and involvement of multiple institutional review boards. Data needs to be publicly available to facilitate training in these complex frameworks. Lastly, for clinical knowledge to disseminate, some form of code sharing is required between academic centers. There can be a number of discrepancies in results which can arise from due to differences in coding structures [4,5]. Overcoming these minor differences which affect algorithm performance can lead to greater consistency in results which can benefit clinical care. Here is a summary of all the studies reviewed in the article (Table 1).

**Table 1:** Summary of Studies Reviewed in the Article 1) CAC: Coronary Artery Calcium 2) CT: Computed Tomography 3) LAA: Left Atrial Appendage 4) CCTA: Coronary Computed Tomography Angiogram 5) CAD: Coronary Artery Disease

Name Of The Study	Tool used	Year of Study	Aim of the Study
Lessman et al [33]	CNN framework	2016	Automated and accurate method to detect CAC^1^
Wolternick et al [26]	CNN Framework	2016	Automated and accurate method to detect CAC^1^
Cano- Espinosa et al [37]	CNN Framework	2018	Generate Agaston score for CAC^1^ burden
Liu et al [39]	FCN	2017	Develop an automated method for Left Atrial Segmentation in CT^2^ volumes
Lopez et al [40]	Deep CNN Framework	2017	Develop Automated and accurate method for aortic thrombus segmentation and quantification
Jin et al [41]	FCN and Conditional Random Field	2018	Develop Automated and accurate method for LAA^3^ segmentation in CT^2^ volumes
Dormer et al [42]	CNN Framework	2018	Develop automated method for heart chamber segmentation
Baskaran et al [29]	U-Net Architecture	2019	Methods for acute and efficient segmentation of cardiovascular structures in CTTA^4^
De Vos et al [43]	Deep Learning Framework	2016	Identify cardiac and aortic regions in 2D images derived from CT^2^ slices and find the corresponding regions on 3D
Moradi et al [44]	CNN Framework and Conditional Random Field	2016	Assess deep learning tool in for semantic labeling of cardiac CT^2^ slices and recognition of body position.
Zreik et al [46]	Deep Learning Model	2018	Assess myocardium in CCTA^4^ and identify patients with functionally significant coronary artery stenosis.
Motwani et al [47]	Deep Learning Algorithm	2016	Predict all-cause mortality in patients who were diagnosed CAD^5^ via CCTA^4^
Commanduer et al [48]	Deep Learning Algorithm	2018	Evaluate epicardial adipose tissue in CT^2^
Gulsun et al [49]	CNN Framework	2016	Method to extract coronary centerlines from CCTA^4^ scans

## Conclusions

Deep learning is not “if” or “how” but an eventual inevitability in the field of CT and cardiovascular imaging. In this dynamic era of big data and collection, pre-existing beliefs and conceptions must evolve for deep learning to be adopted in the medical field. Discoveries need to be data-driven for patient care to prosper. It is natural human instinct to fear change but scientific progress is based on exploring new possibilities. Deep learning can greatly aid cardiovascular imaging by allowing rapid automation and execution of variety of tasks. This can improve medical reasoning and help budding physicians. At the same time, a number of legal, ethical, and social issues must be dealt with. Deep learning is building a bridge between man and machine which will take clinical care to new heights. 
